# Platelet-Rich Gel Supernatants Stimulate the Release of Anti-Inflammatory Proteins on Culture Media of Normal Equine Synovial Membrane Explants

**DOI:** 10.1155/2015/547052

**Published:** 2015-05-18

**Authors:** Diana L. Ríos, Catalina López, Jorge U. Carmona

**Affiliations:** Grupo de Investigación Terapia Regenerativa, Departamento de Salud Animal, Universidad de Caldas, Calle 65 No. 26-10, Manizales, Colombia

## Abstract

The aims were as follows: (1) to evaluate the effects at 48 and 96 h of two concentrations (25 and 50%) of leukocyte and platelet-rich gel (L-PRG) and pure PRG (P-PRG) supernatants on the production/degradation in normal equine synovial membrane explants (SME) of platelet derived growth factor isoform BB, transforming growth factor beta-1, tumor necrosis factor alpha, interleukin (IL-) 4 (IL-4), IL-1 receptor antagonist (IL-1ra), and hyaluronan (HA) synthesis and (2) to correlate these molecules with their respective PRG supernatant treatments. SME from 6 horses were cultured for 96 h with L-PRG and P-PRG supernatants at 25 and 50% concentrations, respectively. SME culture media were changed each 48 h and used for determination by ELISA of the molecules, which were also determined in synovial fluid. 25% L-PRG supernatant produced a sustained release over time of IL-1ra and a gradual release of HA, whereas 50% L-PRG supernatant produced a sustained increase over time of IL-4 and HA. 50% P-PRG supernatant produced an increased and sustained production of IL-1ra and IL-4. The cellular composition and the articular concentration (volume) of a platelet-rich plasma preparation could affect the anti-inflammatory and anabolic joint responses in horses with osteoarthritis.

## 1. Introduction

Osteoarthritis (OA) is a frequent cause of lameness in horses and a potential cause of wastage of valuable animals [1, 2]. This joint disease could appear as a consequence of several predisposing factors, such as repetitive trauma (traumatic arthritis) and synovitis from diverse causes, such as osteochondrosis (OCD) and joint infection [3]. Although OA, in humans is not normally associated with synovitis, this last alteration is a clinical and pathological remark in horses with this pathology [4]. In general, it is well established that synovitis increases the articular cartilage damage by both gene upregulation and production of catabolic cytokines, mainly interleukin-1 (IL-1), tumor necrosis alpha (TNF-*α*), matrix metalloproteinases (MMPs), and eicosanoids, among others [3, 4].

Although equine OA has routinely been treated with intra-articular injection of corticosteroids and hyaluronan [5], there are currently several emerging regenerative therapies, such as autologous conditioned serum (ACS) [6], autologous protein solution (APS) [7], stem cells [8], and platelet-rich plasma (PRP) [9–11]. Notably, PRP could be considered as one of the most worldwide clinical regenerative therapies used in people [12], horses [10, 11], and dogs with OA [13, 14].

Some reports indicate the beneficial effect of PRP in horses with naturally occurring OA [10, 11] and some* in vitro* studies [15, 16] and* in vivo* research [17] have recently been performed in order to explain how PRP could induce articular tissue anabolism. However, there is little information on the basic mechanisms by which this substance produces pain relief and improvement of the joint function.

Currently, there is no consensus on how to employ the “ideal PRP preparation” for the intraarticular joint treatment in patients with OA [16]. Although some ideas have been proposed in order to classify the plethora of PRP preparations used [18], in general, in the horse, these substances could be classified as leukocyte and platelet-rich plasma (L-PRP) and pure platelet-rich plasma (P-PRP). L-PRP preparations show both increased platelet (PLT) (~3–5-fold or more) and leukocyte (WBC) (3-fold or more) counts with respect to basal cell counts in whole blood. P-PRP products show from low physiological to 2-fold PLT counts and from negligible WBC concentration to 2-fold WBC counts with respect to basal cell counts in whole blood [19]. When these PRP preparations are activated, they are transformed in platelet rich gels (PRGs). Thus, PRG from L-PRP is termed L-PRG and PRG from P-PRP is termed P-PRG [18].

As mentioned, synovitis is a* sine qua* nonclinical and pathological alteration in horses with OA [4]. Bearing in mind this, it is necessary to know how supernatants from L-PRG and P-PPG could affect the inflammatory response and metabolism of the equine synovial membrane. Thus, the aims of this research were (1) to evaluate the temporal effects (at 48 and 96 h) of two concentrations (25 and 50%) of L-PRG and P-PRG supernatants on the production or degradation in normal equine synovial membrane explants (SME) of anabolic growth factors (platelet derived growth factor isoform BB (PDGF-BB) and transforming growth factor beta-1 (TGF-*β*_1_)), the proinflammatory tumor necrosis factor alpha (TNF-*α*), anti-inflammatory cytokines interleukin- (IL-) 4 (IL-4) and IL-1 receptor antagonist (IL-1ra), and hyaluronan (HA) and (2) to perform a correlation analysis between these molecules and their respective PRG supernatant treatments.

## 2. Material and Methods

This study was approved by the Ethical Committee for Animal Experimentation of the authors' institution.

### 2.1. Animals and Samples

Synovial membrane samples from the dorsal metacarpophalangeal joints from 6 horses with a mean age of 9 (±3.3) years were included. The samples were from horses free from muscle-skeletal disease and euthanized by a pentobarbital intravenous overdose for other medical reasons. All the joints were radiographed and macroscopically evaluated for excluding horses with OA joint associated changes. Further, 2 mL of synovial fluid were obtained from each joint in order to know the actual concentrations of PDGF-BB, TGF-*β*_1_, TNF-*α*, IL-4, IL-1ra, and HA.

### 2.2. L-PRP and P-PRP Preparation

Venous blood from 1 adult clinically healthy, 11-year-old mare was used to avoid the great variability in the GF, cytokine, and HA concentrations in the PRGs supernatants used in the experiments. L-PRP and P-PRP were obtained by a manual double centrifugation tube method [20], previously validated and used clinically in horses with OA [10]. Briefly, blood was drawn from jugular venipuncture and deposited in 4.5 mL tubes with sodium citrate solution (BD Vacutainer, Becton Drive, Franklin Lakes, NJ, USA). After centrifugation at 120 g for five minutes, the first 50% of the top supernatant plasma fraction, adjacent to the buffy coat, was collected. This fraction was then centrifuged at 240 g for five minutes and the bottom fourth fraction was collected. This fraction was considered L-PRP. The upper plasma fraction was considered as P-PRP. Whole blood and both PRP were analyzed for PLT and WBC concentration using an impedance-based hematology device (Celltac-*α* MEK 6450, Nihon Kohden, Japan).

Both PRP were activated with calcium gluconate (ratio 1 : 10) and remained in incubation at 37°C for 1 h until clot retraction. L-PRG and P-PRG supernatants were always used fresh during each culture media changing at 1 and 49 h. Aliquots of both PRG supernatants obtained at every time point were frozen at −86°C for later quantification of the molecules of interest.

### 2.3. Culture and Study Design

Synovial membrane samples were obtained aseptically and circular 4 mm diameter explants were obtained using a disposable biopsy punch (KAI Medical, Solingen, Germany). SME were dissected from the joint capsule and washed in phosphate buffered saline. The design of the study included the evaluation of five experimental groups, as follows: 1 SME control group (without addition of any PRG supernatant) and 4 SME groups cultured with L-PRG or P-PRG supernatants at two different concentrations, 25% and 50%.

Synovial membrane explants were stabilized in culture media (DMEM, Lonza Group Ltd., Basel, Switzerland) and supplemented with streptomycin (100 *μ*g/mL) and penicillin (100 *μ*g/mL) without the addition of serum. Cultures were incubated in a 5% CO_2_ and water saturated atmosphere for 24 h and then replaced for fresh culture media. After 1 h of incubation L-PRG and P-PRG supernatants were added for obtaining concentrations at 25 and 50%. All SME groups were cultured during 48 h and the culture media were changed and replaced by fresh culture media and fresh PRG supernatants and incubated for other additional 48 h. Culture media obtained at 1, 48, 49, and 96 h were aliquoted and frozen at −86°C for later determination of PDGF-BB, TGF-*β*_1_, TNF-*α*, IL-4, IL-1ra, and HA.

### 2.4. ELISA Analysis

L-PRG and P-PRG supernatants, culture media from SME groups obtained at 1, 48, 49, and 96 h, and synovial fluid were analyzed for measuring (by duplicate) the concentrations of the molecules of interest using ELISA kits (R&D Systems, Minneapolis, MN, USA). PDGF-BB (Human PDGF-BB DuoSet, DY220) and TGF-*β*_1_ (Human TGF-*β*_1_ DuoSet, DY240E) were determined using human antibodies, because there is a high homology between these proteins in humans and horses [21, 22]. In addition, these kits have been used for the same purposes in other equine PRP studies [17, 20]. TNF-*α* (Equine TNF-alpha DuoSet, DY1814), IL-4 (Equine IL-4 DuoSet, DY1809), and IL-1ra (Equine IL-1ra/IL-1F3 DuoSet, DY1814) were assayed with equine specific antibodies and HA (Hyaluronan DuoSet, DY3614) was determined using a multispecies detection ELISA kit. Standards provided for each ELISA kit were used for preparing each standard curve following the manufacturers' instructions. Readings were performed at 450 nm.

### 2.5. Statistical Analysis

The statistical analysis was performed with the software SPSS 19.0 (IBM, Chicago, IL, USA). A Shapiro-Wilk test was used to assess the fit of data set to a normal distribution (goodness of fit). Both PLT and WBC counts in whole blood and both PRP and PDGF-BB, TGF-*β*_1_, TNF-*α*, IL-4, IL-1ra, and HA concentrations in all the evaluated groups showed a normal distribution (*P* > 0.05).

Platelet and WBC counts in whole blood, L-PRP, and P-PRP were evaluated by a one-way analysis of variance (ANOVA), followed by a Tukey test. PDGF-BB, TGF-*β*_1_, TNF-*α*, IL-4, IL-1ra, and HA concentrations from both PRG supernatants, synovial fluid, and blood cells were evaluated in a similar fashion. PDGF-BB, TGF-*β*_1_, TNF-*α*, IL-4, IL-1ra, and HA concentrations from synovial fluid and culture media obtained at 48 and 96 h from all SME groups were analyzed by a generalized lineal model (GLM) followed when necessary by a Tukey test.

PDGF-BB, TGF-*β*_1_, TNF-*α*, IL-4, IL-1ra, and HA concentrations in fresh culture media with PRG supernatants at 1 h and 48 h were also compared with the concentrations for these molecules in the culture media from SME groups obtained at 48 h and 96 h using a *t*-paired test. A correlation analysis was performed to determine the Pearson correlation coefficient (*r*) between the variables evaluated in the study. A *P* < 0.05 value was accepted as statistically significant for all tests. Data are presented as mean ± standard error (s.e).

## 3. Results

### 3.1. Cell and Growth Factor, Cytokine, and HA Concentration in L-PRP/L-PRG, P-PRP/P-PRG, and Synovial Fluid

Platelet counts were significantly (*P* < 0.05_[Tukey  test]_) different between whole blood, L-PRP, and P-PRP, with the lowest concentration for P-PRP (99.4 ± 4.3 PLT/*μ*L (mean ± mean standard error)), followed by whole blood (124.7 ± 3.1 PLT/*μ*L) and L-PRP (311.6 ± 20.4 PLT/*μ*L). WBC counts were also significantly different between the evaluated groups, with a higher concentration for L-PRP (34.2 ± 3.7 WBC/*μ*L), followed by whole blood (8.4 ± 3.6 WBC/*μ*L) and P-PRP (0.13 ± 0.03 WBC/*μ*L).

TGF-*β*_1_ concentration was similar between L-PRG, P-PRG, and synovial fluid. PDGF-BB had a significantly (*P* < 0.05_[Tukey  test]_) higher concentration in L-PRG when compared with P-PRG and synovial fluid; however, the concentration for this GF was similar between these two last components. TNF-*α* concentration was significantly (*P* < 0.01_[Tukey  test]_) higher in synovial fluid when compared to P-PRG supernatant. However, there were not significant differences for this cytokine in supernatants from L-PRG and P-PRG. IL-4 concentration was significantly (*P* < 0.05_[Tukey  test]_) higher in synovial fluid when compared to both PRG supernatants; however, the concentration of this cytokine was similar between both PRG supernatants. IL-1ra concentration was similar between L-PRG supernatant and synovial fluid, but the concentration of this cytokine was significantly (*P* < 0.01_[Tukey  test]_) lower in P-PRG supernatants. HA concentration was significantly (*P* < 0.05_[Tukey  test]_) higher in synovial fluid when compared to both PRG supernatants, but it was similar between both PRG supernatants (Table 1).

### 3.2. Production/Degradation of Growth Factors, Cytokines, and HA in Culture Media of SME TGF-*β*_1_

**Figure 1:**
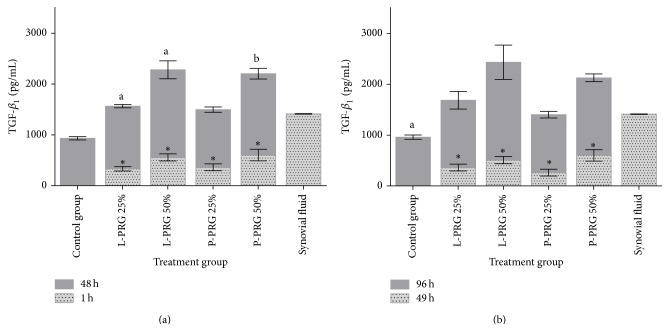
(a) ^a-b^Lowercase letters denote significant (*P* < 0.05) differences between groups in the same column by Tukey test at 48 h. Significantly different with a: L-PRG 50% and P-PRG 50% and b: L-PRP 50%. (b) ^a^Lowercase letters denote significant (*P* < 0.05) differences between groups in the same column by Tukey test at 96 h. Significantly different with a: L-PRG 50%. ^∗^Significant differences (*P* < 0.01) between the same variable at 1 h and 48 h and at 49 h and 96 by *t*-paired test.

Initial TGF-*β*_1_ concentrations obtained at 1 and 46 h in the culture media were significantly (*P* < 0.05_[*t*-paired  test]_) lower when compared with every homologous PRG supernatant treatment at 48 and 96 h, respectively (Figures 1(a) and 1(b)). This GF was substantially produced from SME control group and its concentration at 48 h was similar compared to those TGF-*β*_1_ concentrations measured in the culture media from the SME treated with both 25% PRG supernatants. SME group cultured with 50% L-PRG supernatant presented the highest (*P* < 0.05_[Tukey  test]_) concentration for this protein when compared with the SME control group and those SME groups treated with both 25% PRG supernatants (Figure 1(a)). At 96 h, a significant (*P* < 0.05_[Tukey  test]_) increased TGF-*β*_1_ concentration was observed in SME group cultured with 50% L-PRG supernatant in comparison with SME control group (Figure 1(b)). To note, synovial fluid TGF-*β*_1_ concentration was similar to the culture media from all SME evaluated groups at 48 and 96 h.

### 3.3. PDGF-BB

**Figure 2:**
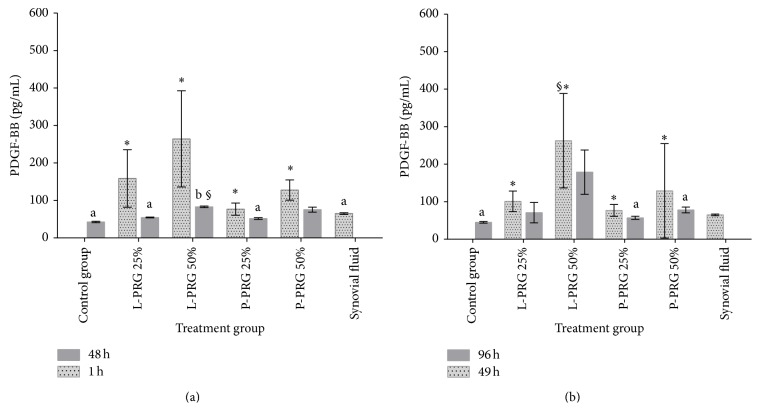
(a) ^a-b^Lowercase letters denote significant (*P* < 0.05) differences between groups in the same column by Tukey test at 48 h. Significantly different with a: L-PRG 50% and P-PRG 50% and b: synovial fluid (SF). (b) ^a^Lowercase letters denote significant (*P* < 0.05) differences between groups in the same column by Tukey test at 96 h. Significantly different with a: L-PRG 50%. ^∗^Significant differences (*P* < 0.01) between the same variable at 1 h and 48 h and at 49 h and 96 by *t*-paired test. ^§∗^Significant differences (*P* < 0.05) between the same variable at 48 and 96 h by Tukey test.

Platelet derived growth factor-BB concentration was significantly (*P* < 0.05_[*t*-paired  test]_) higher in culture media from all SME groups treated with different PRG supernatant concentrations at 1 and 49 h when compared with those PDGF-BB concentrations measured in the same groups at 48 and 96 h, respectively (Figures 2(a) and 2(b)). At 48 h, a significant diminution of PDGF-BB concentration was noticed for all SME groups treated with all PRG supernatants. At this time point, culture media from SME control group presented PDGF-BB concentration similar to those concentrations obtained in synovial fluid and the SME groups treated with both 25% PRG supernatant concentrations. In addition, culture media from SME treated with 50% L-PRG supernatant displayed a significant (*P* < 0.05_[Tukey  test]_) increase of PDGF-BB concentration when compared to the SME control group and SME groups treated with both 25% PRG supernatant concentrations (Figure 2(a)).

At 96 h a similar trend for the concentration of this GF was observed, although PDFG-BB concentration from culture media of the SME treated with 50% of L-PRG supernatant was significantly (*P* < 0.05_[Tukey  test]_) higher when compared with the same group at 48 h (Figure 2(b)). Notably, the concentration for this growth factor was maintained in all the evaluated SME groups in a near or similar concentrations to those obtained in synovial fluid, except for the PDFG-BB concentration from the culture medium of the SME treated with 50% of L-PRG at 96 h.

### 3.4. TNF-*α*

**Figure 3:**
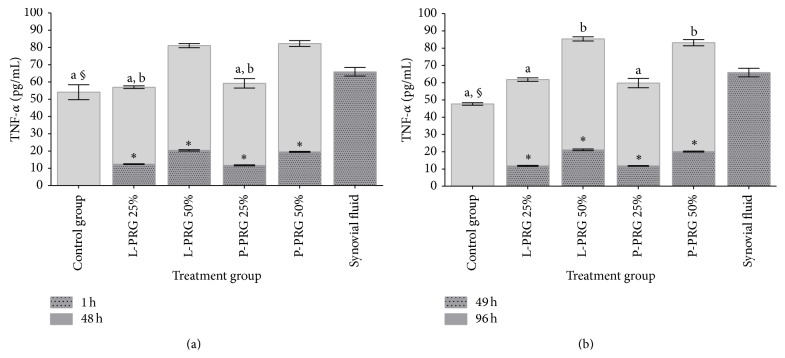
(a) ^a-b^Lowercase letters denote significant (*P* < 0.05) differences between groups in the same column by Tukey test at 48 h. Significantly different with a: SF and b: L-PRG 50% and P-PRG 50%. (b) ^a-b^Lowercase letters denote significant (*P* < 0.05) differences between groups in the same column by Tukey test at 96 h. Significantly different with a: SF and b: control group, L-PRG 25% and P-PRG 25%. ^∗^Significant differences (*P* < 0.05) between the same variable at 1 h and 48 h and at 49 h and 96 by *t*-paired test. ^§^Significant differences (*P* < 0.05) between the same variable at 48 and 96 h by Tukey test.

Tumor necrosis factor alpha was released to the culture media from all SME groups evaluated. At 48 h, the concentration for this cytokine in the SME control group and the groups treated with both 25% PRG concentrations was significantly different (*P* < 0.05_[Tukey  test]_) when compared to synovial fluid. On the other hand, TNF-*α* concentration was significantly (*P* < 0.05_[Tukey  test]_) lower in culture media from SME treated with 25% L-PRG supernatant in comparison with those groups treated with both 50% PRG supernatants (Figure 3(a)). At 96 h, a similar trend in the concentrations for this cytokine in all the evaluated groups was noticed (Figure 3(b)).

### 3.5. IL-4

**Figure 4:**
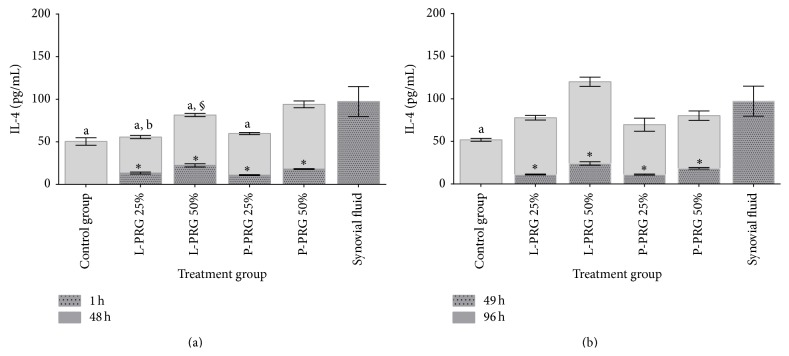
(a) ^a-b^Lowercase letters denote significant (*P* < 0.05) differences between groups in the same column by Tukey test at 48 h. Significantly different with a: SF and b: P-PRG 50%. (b) ^a^Lowercase letters denote significant (*P* < 0.05) differences between groups in the same column by Tukey test at 96 h. Significantly different with a: SF. ^∗^Significant differences (*P* < 0.05) between the same variable at 1 h and 48 h and at 49 h and 96 by *t*-paired test. ^§^Significant differences (*P* < 0.05) between the same variable at 48 and 96 h by Tukey test.

At 48 h, IL-4 concentration was significantly (*P* < 0.05_[Tukey  test]_) lower in culture media from the SME control group and those groups treated with both L-PRG supernatant concentrations and the 25% P-PRG concentration in comparison to SME group treated with 50% P-PRG concentration. IL-4 synovial fluid concentration was similar to those obtained in culture media from SME treated with 50% P-PRG supernatant (Figure 4(a)). At 96 h, IL-4 concentration was similar between synovial fluid and culture media from SME groups treated with PRG supernatants. On contrary, the concentration for this cytokine was significantly (*P* < 0.05_[Tukey  test]_) lower in culture media from SME control group in comparison to synovial fluid (Figure 4(b)). Furthermore, at 96 h, there was a significant increase of IL-4 concentration in the SME treated with 50% L-PRG supernatant in comparison with the culture media from the same group at 48 h.

### 3.6. IL-1ra

**Figure 5:**
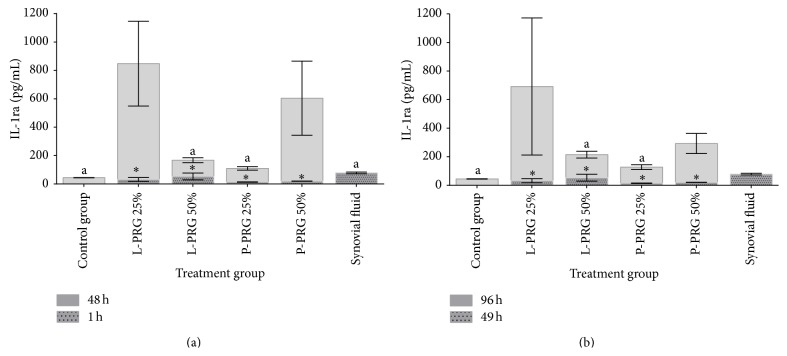
(a) ^a-b^Lowercase letters denote significant (*P* < 0.05) differences between groups in the same column by Tukey test at 48 h. Significantly different with a: L-PRG 25%. (b) ^a^Lowercase letters denote significant (*P* < 0.05) differences between groups in the same column by Tukey test at 96 h. Significantly different with a: L-PRP 25%. ^∗^Significant differences (*P* < 0.05) between the same variable at 1 h and 48 h and at 49 h and 96 by *t*-paired test.

At 48 and 96 h, IL-1ra concentration was significantly (*P* < 0.05_[Tukey  test]_) higher in the SME group treated with 25% L-PRG supernatant in comparison to synovial fluid and the culture media from the SME control group and those groups treated with 50% L-PRG and 25% P-PRG supernatants (Figures 5(a) and 5(b)).

### 3.7. HA

**Figure 6:**
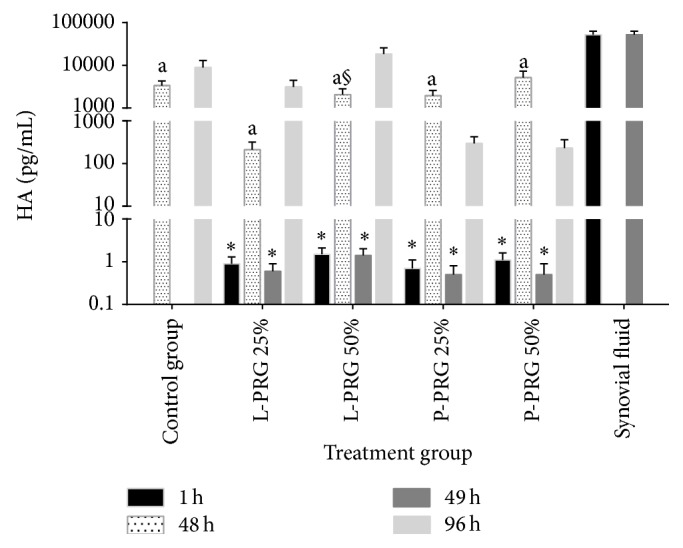
^a^Lowercase letters denote significant (*P* < 0.05) differences between groups in the same column by Tukey test at 48 h. Significantly different with a: SF. ^∗^Significant differences (*P* < 0.05) between the same variable at 1 h and 48 h and at 49 h and 96 by *t*-paired test. ^§^Significant differences (*P* < 0.05) between the same variable at 48 and 96 h by Tukey test.

At 1 and 49 h, HA concentration was significantly (*P* < 0.05_[Tukey  test]_) lower in all culture media of the SME groups treated with PRG supernatants (Figure 6). At 48 and 96 h a significant (*P* < 0.05_[*t*-paired  test]_) increase in the concentration of this molecule was evident in all SME groups evaluated respect to 1 and 46 h. However, HA synovial fluid concentration was significantly (*P* < 0.05_[Tukey  test]_) higher in comparison with the culture media from all SME evaluated groups (Figure 6). At 96 h, there was a significant increased HA concentration in culture medium from SME group treated with 50% L-PRG supernatant in comparison with the HA concentration of the same group at 48 h. Notably, 50% L-PRG supernatant stimulated (although not significantly) the highest HA release to the culture media of the SME treated in comparison with the rest of the SME evaluated (Figure 6).

### 3.8. Correlations

No significant correlations were found between the variables studied.

## 4. Discussion

The present study was aimed at knowing the* in vitro* effect of two PRG supernatants at 25 and 50% concentrations on both production and loss (degradation) in SME of some key molecules closely associated with the pathophysiology of OA [3]. TGF-*β*_1_ and PDGF-BB were evaluated in this research, because they have been demonstrated for their important anabolic effects on joint tissues. In general, both proteins increase ECM cartilage synthesis, diminish both joint pain and inflammation, and promote the differentiation of synovial membrane cells in chondrocytes [23]. Both GF are mainly stored in platelet alpha granules, the reason why many of the therapeutic effects of PRP have been attributed to these proteins [19]. TNF-*α* was selected as a proinflammatory cytokine, because this protein and IL-1 represent the cornerstone proteins associated with the typical catabolic unbalance from OA [24]. This protein is also important in synovial inflammation and its upregulation in synovial tissue is associated with a more aggressive clinical picture of erosive arthritis [25]. In addition, recently a clinical study revealed that TNF-*α* is a useful biomarker for discriminating OA severity in horses in comparison to IL-1 [26].

IL-4 and IL-1ra were chosen because there are important anti-inflammatory cytokines related directly with OA pathophysiology [3]. IL-4 is associated with chondroprotection because it increases the synthesis of ECM cartilage [27]. However, the role for this cytokine in arthritis is more anti-inflammatory than anabolic because it increases the synthesis of IL-1ra and downregulates TNF-*α* [28–30]. On the other hand, IL-1ra is a natural antagonist of IL-1 effects, because it blocks the cellular receptors necessary for inducing joint inflammation and cartilage catabolism mediated by this last cytokine [31].

One of the most important aspects of the present study was that all the molecules evaluated were measured in PRG supernatants, culture media (at different time points), and synovial fluid. This methodological approach allowed establishing that SME groups attempted to reach in their culture media molecular environment to be similar to those observed in synovial fluid samples. However, in general, the SME groups treated with PRG supernatants showed a more robust physiological response related with a higher release to the culture media of all molecules evaluated in comparison with SME control group.

TGF-*β*_1_ and PDGF-BB reached culture media concentrations at 48 and 96 h in a similar pattern to those observed after PRP joint injection in horses at different time points [17]. This could indicate that the synovial membrane cells equilibrate the concentration of TGF-*β*_1_ and PDGF-BB, either by protein production or degradation, in order to reach a natural proportion of these proteins in synovial fluid.

The results from this study allow identifying that both L-PRG supernatants presented better anti-inflammatory and anabolic effects than both P-PRG supernatants. 25% L-PRG supernatant produced a robust and sustained release over time of IL-1ra and a gradual increased release of HA, whereas 50% L-PRG supernatant produced a sustained increase over time in the production of IL-4 and HA. In contrast, 50% P-PRG supernatant produced an anti-inflammatory effect manifested by an increased and sustained production of IL-1ra and IL-4. However, although not significant, the HA concentration tended to diminish over time. Moreover, a 25% P-PRG supernatant could be considered the treatment with the worst results, because it produced the lowest release of anti-inflammatory cytokines and progressive diminution (but not significant) in HA concentration in the time.

The results from this study are contradictory to previous* in vitro* studies evaluating the effect of several PRP preparations on equine cartilage and meniscus explants [15] and equine tendon explants [32, 33]. In general, these studies demonstrated that leukoreduced PRP (P-PRP) preparations produced an anabolic state in these tissues in comparison to L-PRP preparations, which induced upregulation of catabolic molecules, such as matrix metalloproteinases and proinflammatory cytokines [15, 32, 33]. Our findings are not also in agreement with* in vitro* studies that evaluated the effect of PRP preparations on human cartilage explants and synoviocytes in coculture [16] and synoviocytes alone [30].

Some explanations could be proposed to explain the discrepancies in the results from our research and the findings of the aforementioned studies [15, 16, 30, 32, 33]. We believe that different technical and methodological conditions of every study in particular could affect the final results and compromise a direct comparison between all studies. In our study, two concentrations of L-PRG and P-PRG supernatants were evaluated; however, this particular situation was not performed in the mentioned studies [15, 16, 30, 32, 33]. In addition, two culture media changes every 48 h were performed in the present research; in contrast, no culture media changes were performed in these studies [15, 16, 30, 32, 33]. Moreover, we used PRG supernatants for the experiments, whereas PRP preparations were employed in these studies [15, 16, 30, 32, 33].

The results from the present study could indicate that not only the cellular concentration (particularly leukocytes) in PRP but also its concentration on culture media could affect the molecular profile of the tissues treated with this substance. Our findings could also indicate the necessity to establish an adequate volume of PRP or PRG supernatant for the treatment of every specific joint affected by OA or traumatic arthritis. Moreover, these results open the question about which substance, either PRP or PRG supernatant, could be more indicated for OA or traumatic arthritis treatment.

The present study had several limitations. First of all, this was an* in vitro* study, which means that it is only useful for proposing and not for extrapolating the basic mechanisms underlying the PRP based treatment of OA [10–13]. Studies using* in vitro* systems of joint components, like this one, fail to demonstrate the tremendous role of the immunology system and their regulatory action in joint disease [34]. On the other hand, many molecules directly implicated in the OA pathophysiology, such as IL-1, IL-6, and matrix metalloproteinases, amongst others, were not included in this study by budget limitations; this situation logically limits the capacity for understanding the effect of the PRG supernatants evaluated in this study. Finally, further,* in vitro* studies are necessary in order to determine whether the synovial membrane response to PRG supernatants could be affected by inflammation induced by lipopolysaccharide or catabolic cytokines or whether the use of either PRP or PRG supernatant could induce different synovial membrane responses.

## 5. Conclusions

L-PRG and P-PRG supernatants at 25 and 50% concentrations influenced the molecular anti-inflammatory and anabolic profile of SME groups cultured with these substances. Twenty-five % and 50% L-PRG supernatants and 50% P-PRG supernatant produced the best anti-inflammatory and anabolic effects when compared to the SME control group and the SME group treated with 25% P-PRG supernatant. Additional* in vitro* research is necessary to determine if the synovial membrane response to PRG supernatants could be affected by an induced inflammatory state.

## Figures and Tables

**Table 1:** Mean (mean standard error) of the concentration of the molecules evaluated in both leukocyte and platelet-rich gel (L-PRG) and pure platelet-rich gel (P-PRG) supernatants and synovial fluid.

Variable	Fluid		
	L-PRG	P-PRG	Synovial fluid
TGF-*β*_1_ (pg/mL)	1669.2 ± 313.2	1369.2 ± 21.4	1413.8 ± 4.8
PDGF-BB (pg/mL)	3069. 9 ± 1261.6	383.8 ± 80.9^a^	60.5 ± 0.9^a^
TNF-*α* (pg/mL)	60 ± 0.5^a,b^	59 ± 1.4^a^	66.7 ± 3.3^b^
IL-4 (pg/mL)	75.7 ± 9.3^a^	61.1 ± 1.52^a^	101.8 ± 33.7^b^
IL-1ra (pg/mL)	160.4 ± 68.0	58.7 ± 3.1^a^	77.8 ± 10.7
HA (ng/mL)	6.9 ± 2.9	2.3 ± 1.08	53017.6 ± 12140^a^

^a-b^Lowercase letters denote significant differences (*P* < 0.01) between groups in the same column by Tukey test.
